# Maternal Nutrition and Offspring Stress Response—Implications for Future Development of Non-Communicable Disease: A Perspective From India

**DOI:** 10.3389/fpsyt.2019.00795

**Published:** 2019-10-29

**Authors:** Ghattu V. Krishnaveni, Krishnamachari Srinivasan

**Affiliations:** ^1^Epidemiology Research Unit, CSI Holdsworth Memorial Hospital, Mysore, India; ^2^Division of Mental Health and Neurosciences, St. John’s Research Institute, Bangalore, India

**Keywords:** stress response, nutrition, non-communicable disease, cortisol, gestational diabetes

## Abstract

Psychological stress is recognized as a major modifiable risk factor for adult non-communicable disease (NCD) that includes depression, type 2 diabetes mellitus, and cardiovascular disease. Dysregulation of hypothalamic-pituitary-adrenal (HPA) axis activity resulting in either exaggerated or blunted cortisol responses, and altered autonomic cardiovascular control have been thought to underlie this association. The developmental origins hypothesis proposes that impaired nutrition during fetal and early postnatal growth is associated with a higher NCD risk later in life. Maternal nutrients are vital for fetal growth and development, and both maternal undernutrition and over nutrition as in the case of gestational diabetes are associated with higher NCD risk markers in the offspring. Recent studies suggest that fetal exposure to maternal nutritional imbalances may permanently alter cortisol and cardio-sympathetic stress-responsiveness, which may link early life nutrition with adult disease risk. A few recent studies that examined the association between low birth weight as a marker of fetal undernutrition and stress response in humans showed that lower birth weight was associated with an altered HPA axis and cardiovascular sympathetic nervous system responses to stress in adults as well as in children. In addition, altered stress responses in relation to gestational diabetes have been noted. In this paper, we present available evidence from India for the association between maternal nutrition and offspring stress responsiveness against the backdrop of global evidence, and discuss its role in the escalating NCD rates in this population. We also discuss the scope for future studies in India and other transitioning countries.

## Introduction

There has been a steady increase in type 2 diabetes, cardiovascular disease, and other non-communicable diseases (NCD) including depression in the world, but this increase is pronounced in transitioning countries such as India (1). This may be a direct consequence of changing lifestyle behaviors and environmental factors giving rise to increased vulnerability to adiposity and other intermediary phenotypes for NCDs in these populations. Psychological stress is recognized as one such lifestyle-related risk factor (2). Stress is a negative subjective experience that occurs when an individual perceives that the situational demands exceed his/her adaptive capacity (3). Body responds to stress by the activation of the hypothalamic-pituitary-adrenal (HPA) axis (and its end product cortisol) and the autonomic nervous system (ANS), which in turn lead to various physiological changes that help to maintain homeostasis (2). On repeated exposure to stress, a dysregulated HPA axis activity and autonomic cardiovascular control, reflected in either exaggerated or blunted responses of these systems to acute stress has been thought to result in cardio-metabolic, neuro-endocrine, and immunological aberrations responsible for NCDs. Studies in humans show that individuals who show abnormal stress responses are at an increased risk for developing cardiovascular and mental health disorders (4, 5).

It is suggested that a number of biological, environmental, and social factors during different stages of life course may confer variations in stress reactivity among different individuals, and thus susceptibility to future development of diseases (6). Fetal HPA axis is responsive and influenced by external stimuli (7). There is evidence from both human and animal studies that several life style related factors during pregnancy that includes maternal diet, sleep pattern, and physical activity may impact fetal programming through alterations in fetal HPA axis activity (8–10). Among the life style factors, maternal under nutrition during the prenatal period is an important programming signal that has enduring effects on the off spring (11). The current review examines the available evidence linking impaired maternal intra-uterine nutrition with offspring stress responses, with particular emphasis to studies in India which is experiencing an escalating prevalence of NCDs.

### The Developmental Origins of Health and Disease

The Developmental Origins of Health and Disease (DOHaD) hypothesis proposes that impaired nutrition during critical stages of fetal development results in permanent changes in structure and function of key physiological systems (programming) (12). The resulting phenotype offers the maximum survival chances at the available nutrition, by preserving brain growth at the cost of other visceral systems (thrifty phenotype). However, it is proposed that the same phenotype predisposes the individual to insulin resistance, type 2 diabetes, and other NCDs when exposed to “surplus” nutrition in later life. This programming hypothesis often referred to as the Barker hypothesis states that a mismatch between intrauterine and extra-uterine environment results in birth of child that is inadequately adapted for function in the extra-uterine environment and consequently is at risk for later development of chronic medical conditions such as NCDs (13).

The fetal programming hypothesis was initially described by David Barker and colleagues, who showed among UK adults that the prevalence of type 2 diabetes and coronary heart disease was higher in individuals with lower birth weights (14, 15). Similar findings of an association between lower birth weight and a higher risk of coronary heart disease was observed in a study from Mysore, South India (16). Subsequently, these findings were replicated in many different populations.

Particularly, studies from Helsinki Birth Cohort, Finland showed associations of lower size at birth (weight and ponderal index) and at 1 year (weight, height, and body mass index) with a higher prevalence of coronary heart disease (17). A recent study from Helsinki also showed that individuals born small-for-gestational age had significantly higher risk of severe mental disorders during adulthood (18). In another study by *Raikkonen et al.*, adult men who had lower birth size (weight, length, and head circumference) and slower postnatal growth during the first 2 years of life had poorer cognitive abilities in late adulthood (19).

The Dutch Hunger Winter Families studies showed that the individuals exposed to famine conditions *in utero* were more likely to be obese and had a higher prevalence of cardio-metabolic disease and depression (20). Thus, the DOHaD hypothesis now encompasses both maternal undernutrition and overnutrition (for e.g., gestational diabetes mellitus/GDM) as playing a critical role in fetal programming.

### Early Origins of Stress Responses

There is a growing body of evidence from studies in the West that early life factors, including nutrition, may permanently alter stress-responsiveness in humans. This may be an important mechanism in the development of adult chronic diseases. As exemplified by the stress-diathesis model, individuals exposed to similar environmental circumstances react quite differently and central to this paradigm is individual “vulnerability characteristics” that determines a heightened sensitivity to environmental stressors (21). In addition, this heightened sensitivity to environmental stressors operates across life cycle and although rooted in neurobiology and genetic susceptibility, is influenced by developmental processes and life experiences and thus, the consequences for the individual are sustained. From a DOHaD perspective, the individual “vulnerability characteristics” are shaped by both genetics and intrauterine programming.

One of the most studied prenatal factors contributing to altered stress reactivity in children is that of prenatal stress. Prenatal stress refers to an array of affective states that includes distinct but overlapping constructs of anxiety, depression, pregnancy specific anxiety, and response to major life stressors. The effect of maternal depression on altered stress reactivity in children is the best studied. (22). While several studies have shown that exposure to prenatal depression is associated with higher cortisol reactivity (23), blunted HPA axis response and higher systolic blood pressure reactivity (24, 25), others did not report any association between prenatal depression and cortisol reactivity in children (26). A recent review on the association between prenatal depression and altered HPA axis and ANS response in offspring concluded that the evidence is weak (27), but suggested that longitudinal studies of children exposed to prenatal depression may be needed for greater clarity. Anxiety is often co-morbid with depression and maternal cortisol levels are higher in women who have both anxiety and depression compared to women with either anxiety or depression (28). Furthermore, maternal anxiety co-morbid with depression during pregnancy had an additive effect on children’s stress reactivity (29). Others too have noted that prenatal anxiety as opposed to prenatal depression is associated with later adverse child outcomes (30). Recent decades has seen an increasing interest in studying the impact of prenatal stress specific to pregnancy such as anxieties related to labor and delivery on child health outcomes (31, 32). Pregnancy specific anxiety is reported to be associated with altered epigenetic pattern in glucocorticoid receptor in the newborn (33). It is not yet clear whether each of these psychological constructs, which can often co-exist together, confer differential biological risk and thus, results in distinct child health outcomes (34).

### Maternal Nutrition and Stress Responses

Maternal nutrients are vital for the development of the fetus *in utero*. Maternal malnutrition has been shown to be associated with changes in placental morphology and blood flow resulting in inadequate supply of nutrients to the fetus (35). It has been proposed that impaired fetal nutrition alters neuro-endocrine structure and function, and impacts HPA axis feedback systems through glucocorticoid receptors (36, 37), and influences stress reactivity. Specific nutrients in the mother, including B vitamins and choline play a critical role in regulating the expression and functioning of factors related to offspring stress system through epigenetic changes (38, 39). Studies in humans and animals have shown that methylation levels in the promoter regions of the gluco-corticoid receptor and 11β-HSD2 genes may be influenced by maternal undernutrition and/or GDM and have adverse implications for offspring stress responses (40–42). In the following sections we will examine the evidence linking altered maternal nutrition and its impact on biological stress systems in early life.

### Early Nutrition and Stress Responses – Evidence From Animal Models

The bulk of the evidence for the programming effects of maternal undernutrition on HPA axis and ANS comes from animal studies. These studies have shown altered HPA axis functioning both in response to stress and during basal conditions. A study using guinea pigs showed that maternal nutrient restriction during the period of maximal fetal brain growth resulted in altered glucocorticoid receptor expression in fetal brain (43). Subsequently there were sex-specific alterations in glucocorticoid output in the adult offspring (44). Langley-Evans et al. showed that protein restriction in rat dams was associated with changes in several indices of HPA axis activity in the fetus (45). There was increased glucocorticoid receptor binding, and elevated corticosterone-inducible enzymes in higher brain centers, suggesting increased glucocorticoid sensitivity. Undernourishment of pregnant ewes during early gestation has been shown to increase cortisol and sympathetic-adrenal responses to stress in adult sheep offspring (46). In another study, maternal food restriction has been shown to alter HPA axis activity throughout the life course in rat offspring (47). This study showed reduced placental 11b-HSD2 activity and a greater trans-placental transfer of glucocorticoids in relation to severe maternal undernourishment. Further, there was altered stress responsiveness in later life and a state of chronic neuro-endocrinal hyperactivity in adulthood. In a rat model study, adult, non-hypertensive males born to protein-restricted dams were shown to exhibit stress-induced hyper-responsive blood pressure (48). A few animal studies also examined birth weight as a proxy for prenatal undernutrition in relation to indicators of HPA axis functioning. Klemcke et al. reported that low birth weight in pigs following unilateral hysterectomy resulted in a 70% increase in plasma cortisol in the low birth weight piglets (≤1.2 kg) at 3 days of age compared with the “large” birth weight piglets (>1.2 kg) (49).

One study among sheep showed that maternal over nutrition and obesity also influences offspring HPA axis sensitivity and responsiveness (50). Ovine models of pre-pregnancy maternal obesity and nutritional excesses were shown to increase fetal circulating cortisol concentrations compared to controls. Later in adult life, offspring of obese ewes had higher baseline plasma cortisol concentrations, and greater ACTH response to a hormonal challenge. This study gave the first indication of long-term effects of fetal exposure to over nutrition on stress reactivity.

### Evidence From Human Studies

Evidence from human studies for an early programming effect of maternal nutritional status on biological stress systems in off spring, however, has been scarce. Initial studies on these associations examined cortisol levels in non-stressed state and used birth weight as a proxy for intrauterine nutrient deficiencies. In one of the early studies, Phillips et al. examined fasting plasma cortisol concentrations among elderly men born in Hertfordshire in the UK (51). The cortisol values were higher among subjects with a birth weight of 5.5 lb or less compared to those with a birth weight of 5.5 lb or more (408 nmol/L *vs*. 309 nmol/L, respectively). Findings from subsequent studies were inconsistent. However, a meta-analysis of published studies among Caucasian populations until 2004 showed a significant inverse association between birth weight and cortisol concentrations (∼25 nmol/L per kg increase in birth weight) (52). The cortisol concentrations, in turn, were associated with higher systolic blood pressure, higher glucose and triglyceride concentrations, and insulin resistance. It was suggested that these findings reflect an association between lower birth weight and a heightened biological stress response (51). These researchers argued for the need to test dynamic responses to stressful situations to clearly understand the early programming effects.

Further studies in the UK and other parts of Europe did indeed show associations between birth weight and altered HPA axis and cardiac sympathetic stress responses. Wust and colleagues provided the preliminary evidence for associations between birth weight and adrenocortical response (53). In their study, 106 young male twins completed the Trier Social Stress Test (TSST), a standard psychosocial stress paradigm involving free speech and mental arithmetic tasks. This study showed that individuals with lower birth weights had a significantly higher salivary cortisol response to stress. This study expanded the earlier knowledge by showing a consistent effect of birth weight on adrenocortical responses in the face of moderate psychosocial stress. This finding was later replicated in children. Jones et al. observed that lower birth weight was associated with higher cortisol responses to the TSST in 7–9 year old children from Southampton, UK (54). This effect was seen only in boys, whereas in girls an inverse association was observed between birth weight and morning cortisol. Subsequently, *Kajantie et al.* demonstrated among older adults from the Helsinki Birth Cohort that birth weight had an inverse “U” shaped association where both lower and higher birth weights were associated with lower cortisol response to stress. This study suggested that intra-uterine conditions may program not only hyper-responsiveness, but also blunted HPA axis activity (55).

A few studies also observed altered cardiovascular reactivity to stress in relation to fetal growth retardation. One of the first studies of this association among 104 men and 79 women of ∼26 years of age in Australia showed that systolic and diastolic blood pressure and heart rate responses to a standard psychological stress protocol were inversely correlated with birth weight but only in females (56). In contrast, in the study in Southampton children, lower birth weight was associated with greater resting systemic arterial pressure and higher vascular resistance response among boys (57). In girls, lower birth weight was associated with shorter resting pre-ejection period, but there was no association with stress responses. Adding to the evidence on sex-specific programming effects, among 8-year old children in Finland, girls were more likely to have higher systolic and diastolic blood pressure response and overall higher cardiac sympathetic activity in association with lower birth weight (58). They also had slower blood pressure recovery after stress. Boys had overall lower cardiac sympathetic activity.

Thus, though few, human studies have found a consistent association between early nutritional exposures (measured as birth weight) and indices of later neuro-endocrinal stress responses. Furthermore, initial evidence suggests sex-specific programming effects. In a systematic review of the early programming effects on HPA axis authors concluded that there seems to be an increased vulnerability among females particularly in terms of HPA axis reactivity (59). Other investigators exploring the effects of fetal programming on cognitive performance in infants too have observed sex-specific differences in outcome (60, 61). While the biological underpinning of differential sex programming effects are yet to be elucidated, it is suggested that this could be related to sex-dependent differences in placental function and epigenetic mechanisms(62). Furthermore, presence of sex hormones in the developing fetus may be related to the observed sex differences in outcome (61, 63).

### Indian Scenario

India and other south Asian countries are experiencing a phenomenal rise in the prevalence of type 2 diabetes, cardiovascular disease, and other NCDs including depression. Nearly 80 million people in India alone are expected to develop type 2 diabetes by the year 2030 (1). Some investigators have described an Indian phenotype characterized by more total and truncal body fat than Caucasians of similar body weight, and lower lean body mass being linked to a rising prevalence of NCDs in India (64). This “thin-fat” phenotype itself has been thought to result from widespread fetal undernutrition, and recent economic transition resulting in enhanced postnatal growth. The incidence of GDM is also increasing rapidly among urban women, with estimated prevalence of ∼15% currently (65). This, in addition to still widely prevalent maternal nutritional deficiencies, may create a double burden of intra-uterine undernutrition as well as overnutrition resulting in multiple programming effects on the growing fetus (64). However, the effects of early intrauterine nutritional environment on the development of stress mechanisms have been little studied in India.

The only studies in India on programming of stress responses as a risk factor for NCDs come from two birth cohorts in Mysore, India. In preliminary work in Mysore, morning cortisol concentrations were measured in a cohort of 500 adults of 40–60 years of age born in one maternity hospital (66). This study showed that cortisol concentrations were unrelated to birth size, but were strongly positively correlated with cardio-metabolic risk factors including blood pressure, plasma glucose, insulin resistance, and serum triglyceride concentrations. These correlations were stronger than those seen in white Caucasian populations and were amplified by (interacted with) adult adiposity. It was proposed that the high HPA axis activity and maintenance of high cortisol levels in the face of higher adiposity may be an underlying cause for increased cardiovascular risks in south Asian populations.

The above findings were subsequently replicated among the younger participants of Mysore Parthenon cohort (67). Similar to older adults, in the Mysore children (∼9.5 years), higher fasting plasma cortisol was associated with contemporaneous cardio-metabolic risk markers. Unlike in the studies from the west, birth size was not a predictor of fasting cortisol concentrations. However, studies from the West showed that fetal programming effects on HPA axis is more apparent using dynamic stress testing procedures (53–58).

### Maternal Gestational Diabetes Mellitus and Cardiovascular Stress Responses in Indian Adolescents

The above findings prompted the researchers in India to explore the association between maternal nutritional status and offspring stress responses using a dynamic stress paradigm. The only reported study of this association tested the role of intrauterine overnutrition related to maternal GDM in altered offspring stress responses (68). This study was conducted among participants of the Parthenon Cohort, a well-characterized birth cohort at Holdsworth Memorial Hospital in Mysore (69). This cohort was designed to examine the long-term associations of maternal GDM with offspring cardio-metabolic and mental health risks, and provides longitudinal data on this association. Initial investigations of this prospective study had shown that offspring born to GDM mothers were heavier and more adipose at birth, and exhibited greater adiposity, higher insulin resistance, and systolic blood pressure compared to control offspring (offspring born to non-GDM mothers and non-diabetic fathers) during childhood. The associations were less pronounced in offspring of diabetic fathers, thus emphasizing the additional risks associated with intra-uterine exposure to hyperglycemia over and above genetic predisposition.

When the participants were ∼13.5 years of age, the Parthenon study examined their cortisol and cardiovascular responses to acute stress induced by the TSST for children (68). For the test, the adolescents completed 5-min each of a public speaking (imaginative story telling) and a mental arithmetic (serial subtraction) task in front of two unfamiliar adults acting as “judges” (stressor). Salivary samples were collected before and after the stress induction. Cardiovascular parameters were measured continuously before the test during neutral conditions (baseline) and during the stress induction as previously reported. Within the cohort, offspring of GDM mothers exhibited greater systolic blood pressure (5.6 mmHg higher than to controls), cardiac output (0.5 L/min), and stroke volume (4.0 ml) responses and a lower total peripheral response to stress (125 dyn s/cm5) than controls (68). The associations were not strong among offspring of diabetic fathers compared to controls. There was no association of parental hyperglycemia with cortisol responses to stress. The authors hypothesized that stress-response programming may be one of the pathways by which maternal overnutrition increases offspring NCD risk.

### Maternal Micronutrient Status and Autonomic Nervous System Modulation in Children

Autonomic nervous system (ANS) is an important component of bodily stress response system and the HPA axis and ANS are considered as complimentary systems (70). The ANS has two components, the sympathetic and parasympathetic systems, which act in opposite directions. Maternal micronutrient deficiencies, particularly B group vitamins are common among pregnant women in India and studies have noted that maternal vitamin B12 deficiency is associated with adverse offspring outcomes (71, 72). Vitamin B12 plays an important role in myelination and its deficiency could impact myelination of ANS and alter its functioning. Heart rate variability (HRV) is widely used as a non-invasive measure of ANS modulation and is influenced by sympathetic and parasympathetic system (73). While fetal programming of ANS reactivity in children in relation to prenatal stress has been studied (74), few studies have examined the association between maternal nutrient status and ANS reactivity in children. In a study using HRV, severe vitamin B12 deficiency in adults was associated with decreased sympathetic component of ANS (75). A recent study noted that children born to mothers with low maternal vitamin B12 status during pregnancy had reduced cardiac sympathetic activity using indices of HRV (76). In a later study among pregnant women, women in the lowest quartile of vitamin B12 levels had deceased cardiac sympathetic activity as indexed by low frequency component of HRV compared to pregnant women with high vitamin B12 status (77). Reduced availability of vitamin B12 during pregnancy may affect the fetus through its effect on myelination and synaptic connectivity (78) and tissue levels of various neurotransmitters (79). While altered ANS functioning has been shown to be associated with increased risk for cardiovascular conditions in adults (80), it is not yet clear how changes in childhood HRV tracks into adulthood and whether it confers an increased risk for later development of cardiovascular system conditions.

Thus, longitudinal studies are required to fully understand the early programming effects on fetal and childhood ANS modulation and its implications for later development of NCDs.

### Proposed Pathways Linking Prenatal Maternal Nutrition and Altered Stress Reactivity in Humans

A number of mechanisms have been proposed to underlie the early-nutritional programming of NCDs. The growing fetus depends on the mother for its nutritional needs, and it is not surprising, therefore, that any alteration in the maternal nutritional status or its supply to the *conceptus* will have an impact on the optimal fetal growth. It was suggested that when there is a poor nutritional supply, the growing fetus adapts to adverse conditions by prioritizing the growth of the brain, which is vital for survival, thus compromising the growth and functions of “less important” insulin sensitive organs like the pancreas, liver, and skeletal muscles (13). This prioritization could occur by redistribution of blood flow to vital organs, reduced secretion of anabolic hormones such as insulin and insulin-like growth factors (IGF), or increased cortisol production encouraging early differentiation and compromising abdominal visceral and musculo-skeletal growth. It is suggested that these organs “fail” to function in the face of later life metabolic load (e.g., adiposity) leading to disease (81).

Early epigenetic modifications may be another mechanism for early life programming. Epigenetic changes are heritable changes in gene expression without altering DNA sequence. These are established early during fetal growth and are influenced by environmental factors including maternal nutrition and metabolic status (82). Epigenetic modifications mediate differential phenotypic expression of a genotype, and thus may underlie the development of NCDs.

The above pathways may themselves also explain the link between maternal nutritional status and altered stress reactivity. Impaired intrauterine nutrition has been thought to induce permanent changes in the regulation and set point of several hormonal systems (83). In particular, maternal nutrition is thought to alter neuroendocrine structure and function. This has been proposed to impact HPA axis feedback systems through glucocorticoid receptors, and altered sympathetic-adrenal function. This results in altered responses of these systems. Triggering of epigenetic modifications, particularly DNA methylation of glucocorticoid receptors in brain have been thought to result in the persistence of altered functioning of these systems during later life (83). While maternal undernutrition may directly impact fetal programming of stress reactivity systems, it may additionally do so through its association with prenatal stress. It has been suggested that maternal diet during pregnancy may mediate the effects of prenatal stress on fetal programming (8). Others have noted that maternal stress acts as the mediator between dietary deficiencies during pregnancy and programming of the fetus (84) suggesting a bi-directional relationship between maternal stress and maternal nutrient status. Several studies have reported an association between unhealthy diet during pregnancy and maternal depression (85, 86), including increased fat intake in the first trimester (87) and decreased vitamin intake (88). Pregnancy specific anxiety has also been linked to poorer diet quality during pregnancy (89), less vitamin intake (90), and decreased omega 3 fatty acid intake (91). Animal studies have shown that high fat diet is associated with increase in maternal leptin, glucose, insulin, and pro-inflammatory cytokines (92, 93), which can impact fetal neuroendocrine development and HPA axis due to changes in the brain brought on by inflammation (8).

**Figure 1:**
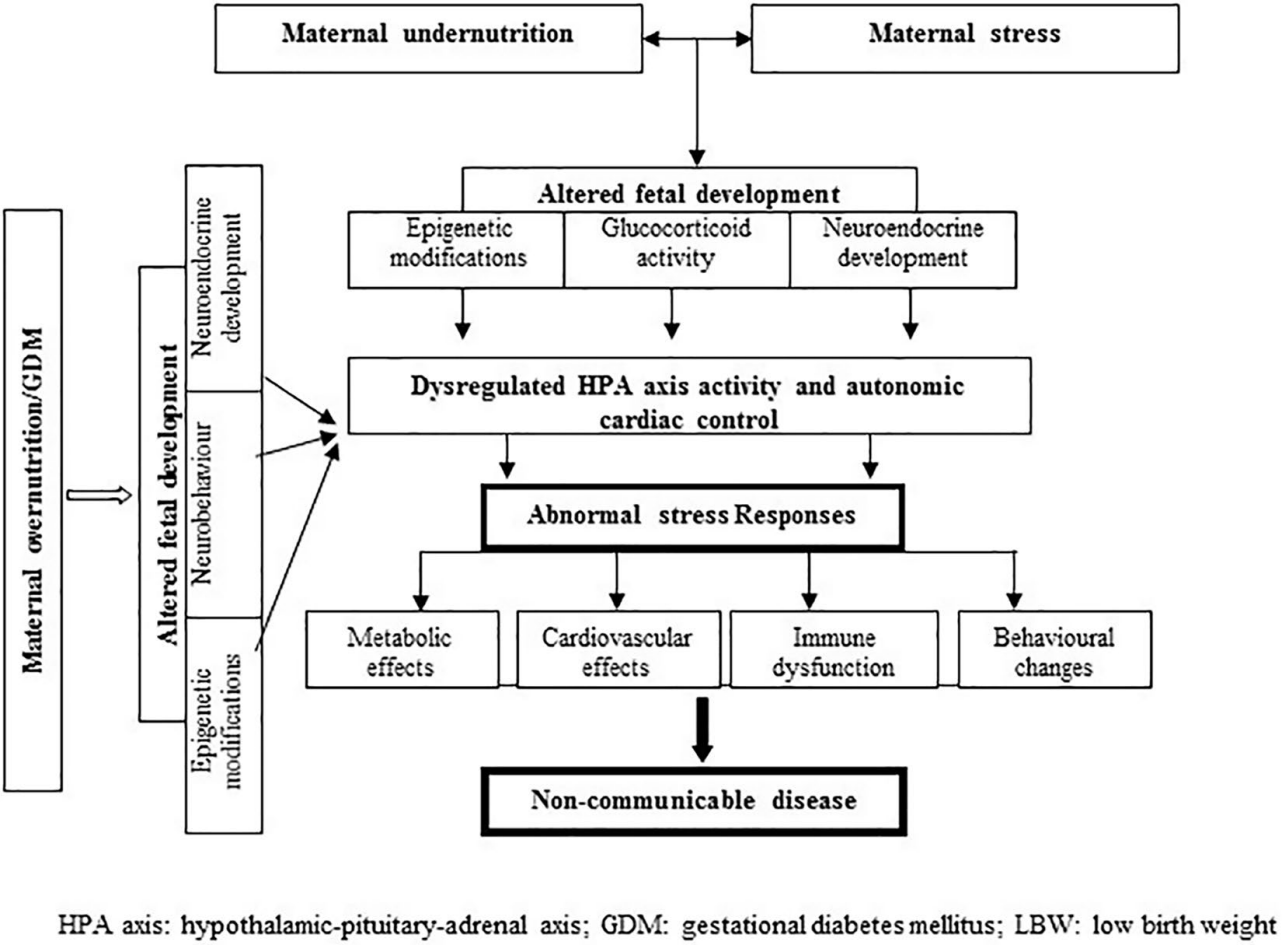
Proposed pathways linking prenatal maternal nutrition and altered stress reactivity in humans.

Mechanism for the association between maternal overnutrition and stress responses is not clear. Animal model studies have shown permanent changes in hypothalamic structure and function in relation to maternal diabetes (94); the stimulatory centers for both HPA axis and sympathetic nervous system are located in the hypothalamus. Maternal GDM may trigger DNA methylation changes as in states of nutritional deficiency (95). Perinatal hyperinsulinemia may also trigger anxiogenic behavior to stress in later life (96). **Figure 1** depicts the pathways described above.

### Gap in Evidence—Future Plans

In India, maternal undernutrition and specific nutritional deficiencies may underlie the high prevalence of intra-uterine growth retardation and childhood undernourishment (97). On top of that, rising prevalence of adiposity and glucose intolerance among young pregnant women is exposing the growing fetus to dual insults of surplus fuel as well as specific nutrient imbalance *in utero*. Research from India and elsewhere suggest that this phenomenon may be a major triggering factor in the rapid escalation in the prevalence of type 2 diabetes and other NCDs in the country. Rapid urbanization in the country has introduced its own challenges in terms of energy-rich diets, decreased scope for physical activity, and increased life stresses, particularly among youth. This may further exacerbate the NCD and adverse mental health situation in the country (98, 99).

In this context, studying stress reactivity as mechanism linking early life nutrition with later development of NCDs, particularly in adolescents and young adults, may provide vital opportunities to intervene during key life course periods. However, despite a growing consensus on the importance of stress systems for later disease development, relationship of maternal nutritional status to offspring stress responses is yet to be clearly elucidated. Maternal nutrients, particularly B-vitamins involved in one-carbon metabolism are known to be vital co-factors in many neuro-developmental and gene methylation processes (100, 101). Findings from a recent study in pregnant African American women showed that fatty acid supplementation resulted in lower cortisol reactivity to a laboratory stressor at 30 weeks of pregnancy (102). Thus, it is imperative that researchers working on DOHaD paradigm, particularly in countries in transition such as India, should embark on studies that clarify the way for future interventions. Specific issues are related to 1) the causal role of maternal nutrition in shaping stress responses in children, 2) mechanism underlying these associations, 3) critical life course periods during which interventions may optimize individuals’ as well as future generation’s stress responses for a better health, and finally 4) identifying suitable interventions for individuals who have already been exposed to sub-optimal nutritional status *in utero*.

These objectives form the premise for a recently launched multi-centric study in India (103). This proposed study aims to examine stress responses in adolescents and young adults in relation to various life course factors including maternal B12, folate and GDM, birth size, and childhood growth. By following up offspring of an existing pre-conceptional intervention cohort, this study provides a unique opportunity to test whether prenatal micronutrient supplementation optimizes physiological stress responses in adolescent children. A range of cardiometabolic parameters, psychological health indicators, and lifestyle factors are being measured. Mechanisms underlying programming of stress responses will be explored through structural brain MRI scans and epigenetics studies. The outcomes of this study are likely to fill some of the gaps identified above. Specifically, this will give robust evidence for the causal role of intra-uterine nutrition in programming stress responses in children and adolescents. Thus, intervention in life style factors that includes maternal diet during pregnancy linked to fetal metabolic programming may be a cost effective way to prevent future development of obesity, type 2 diabetes, and cardiovascular conditions (104).

## Conclusion

In conclusion, it is apparent that, although animal studies have shown consistent association between impaired maternal nutritional status and offspring stress reactivity, research on programming effects among humans is in its early stages. Both animal studies as well as limited evidence in humans suggest that exposure to both undernutrition and overnutrition during fetal development may bring about adverse changes in stress response systems and functioning. This warrants that future studies on stress programming need to focus on both these aspects. Majority of the studies in humans have used birth weight as a proxy for fetal nutrition. However, birth weight is a crude indicator of fetal growth retardation, and does not give a complete estimation of the effects of fetal under nutrition on different physiological systems. Thus measuring offspring stress responses in relation to maternal nutritional status per se will give more objective measures of this association. However, to the best of our knowledge no such studies are available in humans currently, and this is definitely as area for future research. Moreover, causal relationships cannot be inferred by the observational studies and hence prenatal nutritional intervention studies may be an important step forward in this context. More studies adopting a similar approach from different parts of the world may provide robust evidence for the importance of adequate maternal nutrition in shaping optimal stress reactivity throughout life course.

## Author Contributions

Both the authors conceptualized the paper, contributed to the drafting and revising of the manuscript, and read and approved the final content.

## Conflict of Interest

The authors declare that the research was conducted in the absence of any commercial or financial relationships that could be construed as a potential conflict of interest.
